# High-Throughput Phenotyping of Dynamic Canopy Traits Associated with Stay-Green in Grain Sorghum

**DOI:** 10.34133/2020/4635153

**Published:** 2020-09-04

**Authors:** J. D. Liedtke, C. H. Hunt, B. George-Jaeggli, K. Laws, J. Watson, A. B. Potgieter, A. Cruickshank, D. R. Jordan

**Affiliations:** ^1^University of Hohenheim, 70593 Stuttgart, Germany; ^2^Agri-Science Queensland, Department of Agriculture and Fisheries, Warwick, QLD 4370, Australia; ^3^Queensland Alliance for Agriculture and Food Innovation, The University of Queensland, Warwick, QLD 4370, Australia; ^4^Queensland Alliance for Agriculture and Food Innovation, The University of Queensland, Gatton Campus, Toowoomba QLD 4343, Australia

## Abstract

Drought is a recurring phenomenon that puts crop yields at risk and threatens the livelihoods of many people around the globe. Stay-green is a drought adaption phenotype found in sorghum and other cereals. Plants expressing this phenotype show less drought-induced senescence and maintain functional green leaves for longer when water limitation occurs during grain fill, conferring benefits in both yield *per se* and harvestability. The physiological causes of the phenotype are postulated to be water saving through mechanisms such as reduced canopy size or access to extra water through mechanisms such as deeper roots. In sorghum breeding programs, stay-green has traditionally been assessed by comparing visual scores of leaf senescence either by identifying final leaf senescence or by estimating rate of leaf senescence. In this study, we compared measurements of canopy dynamics obtained from remote sensing on two sorghum breeding trials to stay-green values (breeding values) obtained from visual leaf senescence ratings in multienvironment breeding trials to determine which components of canopy development were most closely linked to the stay-green phenotype. Surprisingly, canopy size as estimated using preflowering canopy parameters was weakly correlated with stay-green values for leaf senescence while postflowering canopy parameters showed a much stronger association with leaf senescence. Our study suggests that factors other than canopy size have an important role in the expression of a stay-green phenotype in grain sorghum and further that the use of UAVs with multispectral sensors provides an excellent way of measuring canopy traits of hundreds of plots grown in large field trials.

## 1. Introduction

Sorghum (*Sorghum bicolor* (L.) Moench) is a crop widely grown in drought-prone areas around the world and is mainly used in human and animal nutrition, as fiber or for ethanol production [1]. It is the fifth most important cereal crop in the world and provides food for more than 750 million people in the semiarid tropical regions of Asia, Africa, and Latin America [2]. Population growth in combination with climate change is a challenge for the world's future food security [3]. This demands crops with traits that contribute to tolerance of water deficit. Functional stay-green is a drought adaptation phenotype that is generated by traits that influence water use and water capture and expresses itself in a delayed onset of senescence, a slower senescence rate, or enhanced greenness [4]. Under postflowering drought conditions, stay-green components such as senescence rate and onset of senescence are found to be correlated with increased yield in sorghum [5–7]. Traits that change either the supply or demand for water or the timing of water use all may contribute to a stay-green phenotype. On the supply side, root architectural traits such as longer roots or a narrower root angle can contribute to a greater water extraction from deep in the soil [8, 9]. On the demand site, several studies suggested that a reduced leaf area and enhanced transpiration efficiency play a major role [5, 8, 10–13]. Introgressing stay-green QTLs into a senescent sorghum line resulted in a smaller leaf canopy due to reduced tillering or smaller leaves and led to reduced water use before flowering [8]. Also, crop modelling approaches suggested that in water-limited environments on deep soils with good water-holding capacity, a shift from preanthesis to postanthesis water use via a reduced leaf canopy can lead to higher yields [11, 14]. This is because a smaller canopy uses less water before anthesis and reduces transpiration, and thus, more water is available during grain filling. Where crop growth relies on soil moisture reserves rather than in-crop rain, not only the size of the canopy is an important determinant of water demand but also the leaf area duration, which is mainly driven by phenology. The sooner a hybrid flowers, the more water will remain in the subsoil for the grain-fill period which will affect the expression of stay-green which is why phenology should be considered in the analysis of stay-green. Most of the studies finding an association between stay-green and canopy size are based on a small number of genotypes [8, 10, 12, 13]. In order to use canopy size as a screening trait for stay-green in early-generation variety trials, the association between canopy size and leaf senescence (LSN) needs to be tested across a diverse range of genetic backgrounds and environments, as the expression of a stay-green phenotype may be due to different underlying physiological mechanisms across a range of genetic material [15]. The relative contribution of preanthesis canopy dynamics to stay-green has not been assessed to date. Therefore, the aim of this study was to determine how much attention should be paid to canopy size whilst selecting for genotypes with stay-green-type drought adaptation in sorghum breeding trials. There are a range of approaches available which can be used to measure canopy size. Manual methods for measuring leaf area index with a ruler or leaf area meter are time consuming, expensive, and labor intensive [16], particularly when the number of plots is large. In such cases, the methods preclude measurements at multiple time points making it difficult to capture traits associated with canopy dynamics (rate of canopy development and senescence). Typical plant breeding trials consist of hundreds or thousands of plots, and hence, traits associated with canopy dynamics either are not used in selection or are visually scored. The use of unmanned aerial vehicles (UAV) helps to make the phenotyping process much faster [17, 18] and permits evaluation of canopy size at multiple time points. Remote sensors capture light reflection spectra from plant canopies, which are then used for the calculation of different vegetation indices. A widely used vegetation index is the normalized difference vegetation index (NDVI). The NDVI has been shown to be a good estimator for leaf canopy dynamics and is closely related to LAI [19, 20]. However, when the canopy becomes denser during the vegetative period, the NDVI tends to saturate and underestimate the true LAI [21]. An exponential relationship between the two values can correct for this bias, and an empirical relationship for sorghum has already been developed [22]. Due to this link between canopy size and NDVI, it has also been used to evaluate the stay-green phenotype and its components [23, 24]. Components of the stay-green phenotype, such as high maximum greenness or delayed onset of senescence and rate of senescence and residual “greenness,” can be derived from a logistic [25], linear [26, 27], or polynomial function of NDVI [28] from early crop growth to maturity. Thereby, several sensing metrics can be derived from the functions and correlated with stay-green parameters. These traits include slopes, integrals, and maximum NDVI values of the function. There have not been any previous studies examining the relationship between these traits themselves and their relative importance to stay-green in sorghum. Therefore, the overall objective of this study was to (i) dissect the stay-green phenotype in sorghum into its components using vegetation indices and by that (ii) estimate the influence of canopy size on LSN in multiple environments and a broad range of hybrids and (iii) evaluate the feasibility of using UAV-based sensors to select for stay-green.

## 2. Materials and Methods

### 2.1. Genetic Material

The trials in this study comprised experimental and control hybrids with a total of 427 hybrids grown in Jimbour (26.9627°S, 151.2174°E) and 422 hybrids grown in Pirrinuan (27.0657°S, 151.2653°E). Both are localities of the Western Downs region in Queensland, north-east Australia. Experimental hybrids were obtained through previous crosses of inbred lines with two different female testers from a prebreeding program of the University of Queensland and the Queensland Department of Agriculture and Fisheries. The female parents (testers) were selected for performance across several years and locations. Further, they were deliberately selected for their contrasting stay-green characteristics to expose variation in stay-green expression in the male parents in both a high (Female 1) and a moderate stay-green (Female 2) background.

### 2.2. Experimental Setup and Data Collection

The UAV image data used for this analysis came from two hybrid sorghum breeding trials planted at two different locations in south-east Queensland, Australia. The trials ran from November 2018 to March 2019. The two locations had deep soils with high water-holding capacity. The soils were close to field capacity around planting but thereafter received little or no rainfall. This resulted in typical postflowering drought conditions where the stay-green trait was expressed (i.e., severe enough that senescence due to water limitation occurred across all genotypes). Temperatures were very similar in Jimbour and Pirrinuan at 24.4°C ± 6°C. The experiments in both locations were arranged in partially replicated designs [29] with 39% of the hybrids replicated. Visual LSN scores were taken at plant maturity ranging from 1 to 9, where 1 corresponded to no LSN and 9 to a fully senescent plant canopy. Spectral data was collected via a Tarot custom-made drone with 3DR-Px4 flight controller equipped with a RedEdge multispectral camera (RedEdge, MicaSense, Seattle, Washington) at seven time points during the vegetation period in both trials. Flight altitudes were adjusted depending on plant size and plot cover to ensure sufficient resolution, which resulted in heights of 16, 20, 25, 30, 30, 30, and 35 m from the first to the last flight. Measurements were taken under clear sky conditions on 13 November, 4 December, 19 December, 3 January, 16 January, 12 February, and 28 February/1 March for Jimbour and Pirrinuan, respectively. Once grains on main and tiller panicles were fully matured and sufficiently dry, the trial plots were harvested with a small-plot combine harvester to determine grain yield.

### 2.3. Calculation of Canopy Traits Related to Stay-Green

For the calculation of canopy traits related to stay-green from multispectral data, the free statistical software R (R Development Core Team, 2012) was used. The NDVI index was calculated as an average per plot from the spectral data using the formula

(1) $$\begin{array}{c} \text{NDVI}=\frac{\text{NIR}-\text{red}}{\text{NIR}+\text{red}}. \end{array}$$

For each trial plot, NDVI during the vegetation period was plotted against thermal time. Thermal time from emergence was calculated by calculating 3-hourly averages of daily temperature and accumulating thermal time according to equations (2)–(4) derived from the method of Jones and Kiniry [30].

(2) $$\begin{array}{c} \delta \text{TT}=0\;T<T_{b}\text{or }T>T_{\max}, \end{array}$$

(3) $$\begin{array}{c} \begin{array}{cc} \delta \text{TT}=T-T_{b} & T_{b}<T<T_{\text{opt}} \end{array}, \end{array}$$

(4) $$\begin{array}{c} \delta \text{TT}=\left(T_{\text{opt}}-T_{b}\right)\left[1-\frac{T-T_{\text{opt}}}{T_{\max}-T_{\text{opt}}}\right]T_{\text{opt}}<T<T_{\max}, \end{array}$$

where TT is the thermal time and *T* is the average temperature in each 3-hour period. Base (*T*_*b*_), optimum (*T*_opt_), and maximum (*T*_max_) temperatures were set to 10°C, 30°C, and 42°C, respectively, as used for grain sorghum in Hammer et al. [31].

**Figure 1:**
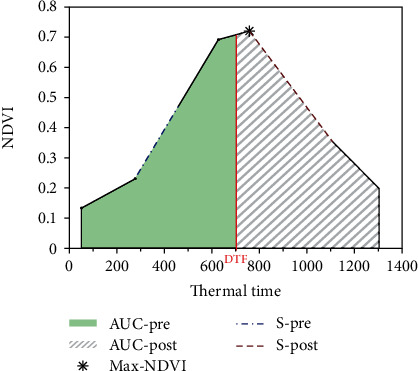
Canopy traits related to stay-green derived from the NDVI curve on an individual plot in Jimbour. Days to flowering for each plot were used as separator between the left and right sides of the curve. Integrals were estimated using the composite trapezoid rule. Traits, their corresponding abbreviation, relevance to stay-green parameters, and units are displayed in Table 1.

The resulting curves were divided into five different components that relate to stay-green parameters: area under the curve preanthesis (AUC-pre), area under the curve postanthesis (AUC-post), slope preanthesis(S-pre), slope postanthesis (S-post), and maximum NDVI value (Figure 1).

To check whether reflectance from bare soil had an effect on the results, the components were also calculated by filtering NDVI values greater than 0.5. When using the filtered NDVI values, the relationships between the components and significance with LSN were essentially the same, requiring no further use of filtered values for the analysis.

### 2.4. Statistical Analysis

#### 2.4.1. Leaf Senescence

Leaf senescence in sorghum is a trait known to show low levels of crossover GxE [7]. Even in the case of small significant GxE interactions, the genotype rankings did not change, which justifies the calculation of across-site LSN BLUPs for each hybrid. For these overall LSN values, a multienvironmental analysis (MET) was conducted including 12 environments using the methods described in Smith et al. [32]. As not all hybrids were measured in all 12 environments, their site-specific LSN values were predicted within the MET based on the environmental main effect in the missing location and their relative sensitivity to environmental changes [33].

#### 2.4.2. Canopy Traits Related to Stay-Green

Because of the potential for phenology to confound estimates of LSN, only individual hybrids that flowered within a five-day flowering window were included in the analysis. After filtering for flowering and excluding commercial varieties, 431 experimental hybrids remained in the dataset of which 251 hybrids were with Female 1 and 180 hybrids were with Female 2 and 133 of the males were common between the two sets. For the canopy components, a joint analysis of both locations was conducted and a linear mixed model was fitted for every trait using the ASReml program [34] inside the statistical package R [35]. There were two different models. One for the average effect of the hybrids and another model considering the population structure of the two different female parents. The basic model for the hybrid effect contained fixed effects for location, plant establishment, and day of flowering. Genotype, replicates, rows, and columns were fitted as random effects. A second model considered the population structure and had an additional fixed effect for the females and a random interaction term for males and females. A first-order autoregressive structure for rows and columns was added in both models to account for spatial correlations. Individual adaptions for each model were made based on a Wald chi-squared test for fixed effects and *z* ratios for random effects, where nonsignificant terms were omitted. In addition, possible linear or spline trends along rows and columns were added if necessary. Best linear unbiased predictors (BLUPS) were calculated for the hybrids in the first model and for males within females in the second model. Furthermore, further BLUPS of the pre- and postanthesis parameters were calculated whilst using maximum NDVI as a covariate in the mixed model for a better separation of each component effect. To check if there were significant correlations between predicted LSN and canopy traits, *t*-tests were applied on a simple linear regression with LSN as the response. Significance for across-site LSN and canopy traits was additionally checked in the ASReml model where the trait was fitted as a fixed effect. Broad-sense heritabilities were calculated as proposed by Cullis et al. [29]. All analyses were done using the statistical package R [35]. A principal component analysis with the BLUPS of all traits for all hybrids was created using the princomp function. Pearson's correlation matrix was calculated using the function “cor.”

## 3. Results

### 3.1. Summary Statistics

**Figure 2:**
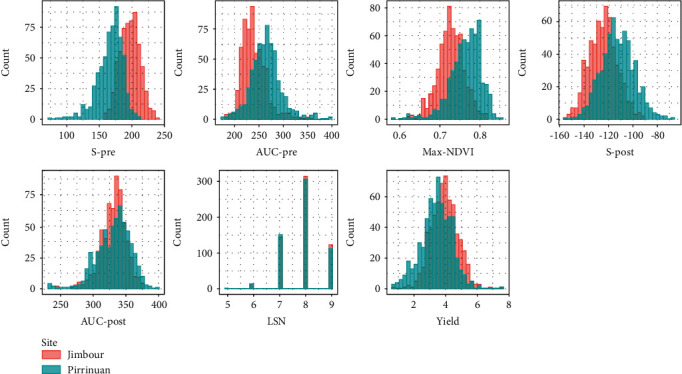
Histograms of canopy traits using raw data of Jimbour and Pirrinuan.

All calculated canopy traits showed moderate to high broad-sense heritabilities in both environments ranging from 37.6 to 92.5 (Table 2). Similar heritabilities have been found for max-NDVI and postflowering parameters in wheat [25, 27]. The heritabilities and genotypic variation for most of the traits were lower in Jimbour than in Pirrinuan. Phenotypic values of canopy traits varied similarly across experiments with higher means in Pirrinuan for most traits (Figure 2). In general, the postanthesis parameters expressed more genotypic variation and higher heritabilities than their preanthesis counterparts. Moreover, across-site LSN values were highly correlated with site-specific LSN values in all environments ranging from 0.54 to 0.99 (data not shown).

### 3.2. Overall Effect of the Hybrids on Canopy Traits Related to Stay-Green

BLUPs of yield and LSN values showed a weak but significant negative correlation (-0.18). Looking at the correlations for the parameters calculated without using max-NDVI as a covariate (SR and AUC-post), the hybrids' across-site LSN values and postanthesis parameters were strongly negatively correlated (Table 3). In comparison to the postanthesis parameters, the preanthesis parameters SL, AUC-pre, and max-NDVI were weakly correlated with across-site LSN (0.05, 0.11, and 0.07, respectively). Moreover, even though max-NDVI itself did not correlate strongly with across-site LSN, it had strong associations with the other components, whereas the correlations of max-NDVI with the preanthesis parameters were higher than those with the postanthesis parameters. Interestingly, AUC-pre and AUC-post were positively correlated whereas S-pre and S-post were showing the opposite relationship. On the other hand, when including max-NDVI as a covariate in the model, the correlation between adjusted AUC-post and adjusted AUC-pre became negative (-0.23). In addition, the previous positive relationship between max-NDVI and AUC-pre became negative, whilst the correlation of across-site LSN with AUC-pre tended toward zero. Moreover, when using max-NDVI as a covariate, the correlation between AUC-post and across-site LSN resulted in a much stronger association as compared to the previous model. As expected, the correlations of AUCs and max-NDVI were substantially reduced. Furthermore, the variation in pre- and postflowering slopes between the different genotypes became negligible.

**Figure 3:**
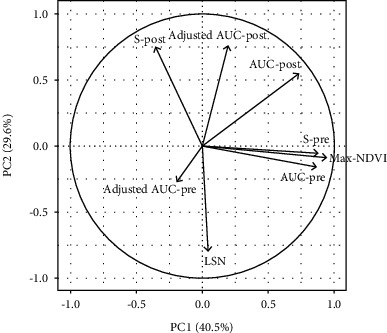
Biplot of calculated canopy traits related to stay-green and leaf senescence (LSN). S-pre, AUC-pre, adjusted AUC-pre, and max-NDVI refer to parameters of canopy size before and around flowering. S-post, AUC-post, and adjusted AUC-post refer to canopy size parameters after flowering.

The biplot of the principal component analysis confirmed a close relationship of postflowering rather than preflowering canopy traits with LSN (Figure 3). The increase in the correlation between LSN and postflowering canopy traits when the postflowering canopy traits were adjusted using max-NDVI as a covariate can be seen clearly. Even though there seemed to be an increase in correlation of LSN and AUC-pre after the adjustment, the substantially reduced loading led to a smaller association.

**Figure 4:**
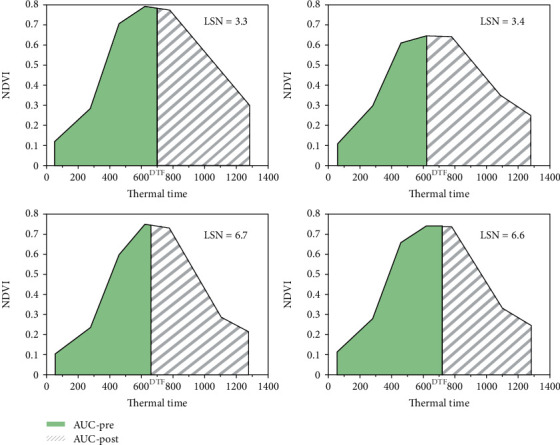
Example plots of sorghum hybrids in Jimbour. AUC-pre: area under the curve preanthesis; AUC-post: area under the curve postanthesis; DTF: days to flowering which were used as a separator between the left and right areas under the curve. The upper plots show low leaf senescence (LSN) values and the lower plots show high LSN values.

Figure 4 shows some example plots from the trial in Jimbour. The upper two panels display plots with low LSN values while the lower two panels show plots with high values. Preanthesis leaf canopy size can be either large or small with no clear difference between high or low senescing plots. On the other hand, postanthesis canopy size seemed to be slightly larger for plots expressing low senescing values.

### 3.3. Effect of Female Parent on Canopy Traits Related to Stay-Green

Comparing the hybrids derived from the two contrasting female testers, differences could be found in their overall LSN as well as in their canopy traits. As expected, hybrids derived from Female 1 (the stay-green female tester) had significantly lower LSN values than those from Female 2 (*P* < 0.001; data not shown). Among the hybrids derived from Female 1, none of the preflowering leaf canopy traits were significantly correlated with LSN (Table 4). All canopy traits were more strongly correlated with yield among the hybrids derived from Female 1, and associations of postanthesis traits with LSN were also greater than among the hybrids derived from Female 2 (the senescent female). Among the hybrids derived from Female 2, preflowering leaf canopy traits were more strongly correlated with LSN of which the correlation with max-NDVI was significant (Table 5). Moreover, the preanthesis traits among the hybrids derived from Female 2 showed higher correlations with the postanthesis traits S-post and AUC-post. In general, it seemed that the preflowering canopy size traits among the hybrids derived from the senescent female had a greater impact on canopy stay-green traits than those among the hybrids derived from the stay-green female.

## 4. Discussion

The aim of this study was to estimate the relative importance of leaf area before anthesis in stay-green phenotypes using NDVI to assess canopy characteristics associated with stay-green.

### 4.1. The Role of Preanthesis Canopy Parameters on the Expression of a Stay-Green Phenotype

In this experiment, preflowering canopy size was uncorrelated or very weakly correlated with LSN. These findings contrast with results found in experiments with wheat where maximum NDVI and NDVI values around maturity were significantly correlated in a drought environment [25]. Also, in sorghum, other canopy traits such as number of tillers or leaf size have been found to be closely linked to stay-green in smaller sets of lines and near isogenic lines [8, 10, 12, 13]. Most likely, the different findings are a result from the broader range of germplasm used in this study and suggest that traits influencing water capture or water use efficiency may play a greater role in the expression of the stay-green phenotype in this material than maximum canopy size (i.e., size of the canopy before flowering). Similarly, in a previous study, the introgression of stay-green QTLs into different genetic backgrounds did cause reduced tillering, leaf area reduction, and a lower maximum leaf area around anthesis in only one of the two QTL introgression lines [15]. This experiment highlighted the importance of genetic background effects, with canopy size being of different importance in the two females. Furthermore, in the hybrids derived from the female with higher LSN, maximum NDVI seemed to be a driver for the correlation between canopy size and LSN. The greater influence of maximum NDVI in hybrids with the senescent female could be related to the relative importance of different component traits that contribute to stay-green. For the stay-green female, traits other than canopy size, for example, root architectural traits, might overshadow any contribution of leaf area differences to the stay-green phenotype. Conversely, in the senescent female, lower water extraction or water use efficiency capacities could lead to an increased role of preflowering leaf canopy parameters to keep the plant green and would therefore explain the significant correlations of max-NDVI and LSN. The adjusted AUC-pre on the other hand did not seem to have any effect on LSN. This might have to do with the average daily water use of sorghum which has its maximum demand during flowering [36]. Therefore, in genotypes where canopy size has an effect on LSN, a reduced canopy size at the time of highest water demand may have a larger impact on the stay-green phenotype than the rate at which the canopy size increased. However, preanthesis canopy traits have a small, context-sensitive effect on stay-green when looking at the range of diverse hybrids evaluated in this study. This indicates that more emphasis should be placed on other component traits by breeders aiming to enhance stay-green drought adaptation.

### 4.2. The Role of Postanthesis Canopy Traits on the Expression of a Stay-Green Phenotype

Postanthesis canopy traits were strongly correlated with overall LSN values (breeding values) derived from multienvironment ratings, and these correlations were even stronger when the data was normalized for canopy size at anthesis by using max-NDVI as a covariate. Postanthesis canopy parameters have also been highly correlated with stay-green ratings in other studies in wheat and sorghum [19, 24, 25]. Postanthesis NDVI not related to canopy size differences indicates increased leaf “greenness” and therefore delayed or slower senescence. This, in turn, is likely driven by greater water uptake or increased water use efficiency. In other studies with sorghum, QTLs for nodal root angle which likely affect water uptake at depth were found to collocate with stay-green QTL [9]. Stay-green has also been associated with increased transpiration efficiency, although those effects have been found to be either context dependent or relatively small [14, 15].

## 5. Conclusion

Within the large set of diverse hybrids observed in this study, it appears that canopy size before flowering made a relatively small contribution to the expression of a stay-green phenotype after flowering. However, the effect varied depending on the female tester which shows the importance of considering genotypic background and other context dependencies when evaluating traits for the selection of complex traits such as stay-green. If stay-green is a result of higher water use during grain filling, traits such as water extraction efficiency and water use efficiency rather than leaf area before flowering may be the main drivers for the expression of the trait. In conclusion, this study showed that variation in canopy size before flowering is not a good predictor of stay-green expression in this set of breeding trials. This result is in contrast to previous observations in smaller genetically less diverse sets of material. In contrast, using UAVs to monitor the NDVI decay after flowering is a suitable method for high-throughput phenotyping of stay-green.

## Figures and Tables

**Table 1:** Abbreviations, relevance to stay-green parameters, and units of calculated traits.

Abbreviation	Trait	Indicator for	Unit
S-pre	Slope preanthesis	Rate of canopy development/green up	(ΔNDVI/Δthermaltime) × 100000
AUC-pre	Total area under the curve preanthesis	Total canopy size before flowering	NDVI × thermaltime
Adjusted AUC-pre	Area under the curve preanthesis with maximum NDVI as covariate	Early canopy size before flowering excluding variances of maximum greenness at flowering	NDVI × thermaltime
Max-NDVI	Maximum NDVI	Maximum canopy size (around anthesis)	Dimensionless
S-post	Slope postanthesis	Rate of senescence	(ΔNDVI/Δthermaltime)∗100000
AUC-post	Total area under the curve postanthesis	Total canopy size after flowering	NDVI × thermaltime
Adjusted AUC-post	Area under the curve postanthesis with maximum NDVI as covariate	Canopy size after flowering excluding variances of maximum greenness at flowering	NDVI × thermaltime

**Table 2:** Summary statistics for the calculated canopy traits and yield (t/ha) at the two locations Jimbour and Pirrinuan.

Trait	Site	Mean	Stde	Gvar	H^2^
S-pre	Jimbour	196	5.6*e*^−04^	3.3*e*^−03^	63.1
	Pirrinuan	165	9.2*e*^−04^	7.5*e*^−03^	83.5
AUC-pre	Jimbour	237	5.95	48.1	54.6
	Pirrinuan	262	10.8	77.0	71.6
Adjusted AUC-pre	Jimbour	237	1.96	12.1	37.6
	Pirrinuan	262	3.88	27.5	72.6
Max-NDVI	Jimbour	0.72	5.1*e*^−04^	4.2*e*^−03^	73.1
	Pirrinuan	0.76	4.2*e*^−04^	3.2*e*^−03^	64.4
S-post	Jimbour	-123	6.1*e*^−04^	6.4*e*^−03^	92.5
	Pirrinuan	-111	5.3*e*^−04^	5.4*e*^−03^	91.0
AUC-post	Jimbour	332	9.15	101.63	78.0
	Pirrinuan	333	11.87	127.47	82.4
Adjusted AUC-post	Jimbour	332	3.21	39.0	76.1
	Pirrinuan	333	6.78	79.8	83.3
LSN	Jimbour	7.86	0.03	0.31	75.1
	Pirrinuan	7.83	0.03	0.27	71.9
Yield	Jimbour	3.95	0.04	0.18	40.7
	Pirrinuan	3.40	0.05	0.21	49.1

Stde: standard error; Gvar: genetic variation; H^2^: broad-sense heritability. Means refer to the raw data.

**Table 3:** Correlation table of the canopy traits (BLUPS) for the average effect of all hybrids.

Trait	S-pre		AUC-pre		Adjusted AUC-pre		Max-NDVI		S-post		AUC-post		Adjusted AUC-post		LSN		Yield	
	Cor.	Sig.	Cor.	Sig.	Cor.	Sig.	Cor.	Sig.	Cor.	Sig.	Cor.	Sig.	Cor.	Sig.	Cor.	Sig.	Cor.	Sig.
S-pre	1	—	0.73	∗∗∗	-0.09	ns	0.79	∗∗∗	-0.34	∗∗∗	0.57	∗∗∗	0.10	ns	0.05	ns	0.13	∗∗
AUC-pre	0.73	∗∗∗	1	—	0.31	∗∗∗	0.80	∗∗∗	-0.40	∗∗∗	0.51	∗∗∗	0.01	ns	0.11	∗	0.25	∗∗∗
Adjusted AUC-pre	-0.09	ns	0.31	∗∗∗	1	—	—	—	0.03	ns	-0.35	∗∗∗	-0.23	∗∗∗	0.07	ns	0.06	ns
Max-NDVI	0.79	∗∗∗	0.80	∗∗∗	—	—	1	—	-0.45	∗∗∗	0.72	∗∗∗	—	—	0.07	ns	0.19	∗∗∗
S-post	-0.34	∗∗∗	-0.4	∗∗∗	0.03	ns	-0.45	∗∗∗	1	—	0.22	∗∗∗	0.73	∗∗∗	-0.69	∗∗∗	0.17	∗∗∗
AUC-post	0.57	∗∗∗	0.51	∗∗∗	-0.35	∗∗∗	0.72	∗∗∗	0.22	∗∗∗	1	—	0.77	∗∗∗	-0.46	∗∗∗	0.33	∗∗∗
Adjusted AUC-post	0.10	ns	0.01	ns	-0.23	∗∗∗	—	—	0.73	∗∗∗	0.77	∗∗∗	1	—	-0.72	∗∗∗	0.30	∗∗∗
LSN	0.05	ns	0.11	∗	0.07	ns	0.07	ns	-0.69	∗∗∗	-0.46	∗∗∗	-0.72	∗∗∗	1	—	-0.18	∗∗∗
Yield	0.13	∗∗	0.25	∗∗∗	0.06	ns	0.19	∗∗∗	0.17	∗∗∗	0.33	∗∗∗	0.30	∗∗∗	-0.18	∗∗∗	1	—

Cor.: correlation; sig.: significance. Significance tested in a regression analysis. Significance level: ^∗∗∗^*P* < 0.001, ^∗∗^*P* < 0.01, ^∗^*P* < 0.05, ns: *P* > 0.05. S-pre: slope preanthesis; AUC-pre: total area under the curve preanthesis; adjusted AUC-pre: adjusted area under the curve preanthesis for maximum greenness; max-NDVI: maximum normalized difference vegetation index; S-post: slope postanthesis; AUC-post: total area under the curve postanthesis; adjusted AUC-post: adjusted area under the curve postanthesis for maximum greenness; LSN: leaf senescence.

**Table 4:** Correlation table of canopy traits (BLUPS) for the average effect of the hybrids with Female 1.

Trait	S-pre		AUC-pre		Adjusted AUC-pre		Max-NDVI		S-post		AUC-post		Adjusted AUC-post		LSN		Yield	
	Cor.	Sig.	Cor.	Sig.	Cor.	Sig.	Cor.	Sig.	Cor.	Sig.	Cor.	Sig.	Cor.	Sig.	Cor.	Sig.	Cor.	Sig.
S-pre	1	—	0.78	∗∗∗	-0.05	ns	0.82	∗∗∗	-0.35	∗∗∗	0.57	∗∗∗	0.10	ns	0.04	ns	0.24	∗∗∗
AUC-pre	0.78	∗∗∗	1	—	0.31	∗∗∗	0.84	∗∗∗	-0.4	∗∗∗	0.54	∗∗∗	0.05	ns	0.05	ns	0.25	∗∗∗
Adjusted AUC-pre	-0.05	ns	0.31	∗∗∗	1	—	—	—	0.02	ns	-0.27	∗∗∗	-0.19	∗∗	0.05	ns	0.04	ns
Max-NDVI	0.82	∗∗∗	0.84	∗∗∗	—	—	1	—	-0.44	∗∗∗	0.71	∗∗∗	—	—	0.05	ns	0.25	∗∗∗
S-post	-0.35	∗∗∗	-0.4	∗∗∗	0.02	ns	-0.44	∗∗∗	1	—	0.24	∗∗∗	0.75	∗∗∗	-0.69	∗∗∗	0.26	∗∗∗
AUC-post	0.57	∗∗∗	0.54	∗∗∗	-0.27	∗∗∗	0.71	∗∗∗	0.24	∗∗∗	1	—	0.78	∗∗∗	-0.47	∗∗∗	0.46	∗∗∗
Adjusted AUC-post	0.10	ns	0.05	ns	-0.19	∗∗	—	—	0.75	∗∗∗	0.78	∗∗∗	1	—	-0.7	∗∗∗	0.46	∗∗∗
LSN	0.04	ns	0.05	ns	0.05	ns	0.05	ns	-0.69	∗∗∗	-0.47	∗∗∗	-0.70	∗∗∗	1	—	-0.33	∗∗∗
Yield	0.24	∗∗∗	0.25	∗∗∗	0.04	ns	0.25	∗∗∗	0.26	∗∗∗	0.46	∗∗∗	0.46	∗∗∗	-0.33	∗∗∗	1	—

Cor.: correlation; sig.: significance. Significance tested in a regression analysis. Significance level: ^∗∗∗^*P* < 0.001, ^∗∗^*P* < 0.01, ^∗^*P* < 0.05, ns: *P* > 0.05. S-pre: slope preanthesis; AUC-pre: total area under the curve preanthesis; adjusted AUC-pre: adjusted area under the curve preanthesis for maximum greenness; max-NDVI: maximum normalized difference vegetation index; S-post: slope postanthesis; AUC-post: total area under the curve postanthesis; adjusted AUC-post: adjusted area under the curve postanthesis for maximum greenness; LSN: leaf senescence.

**Table 5:** Correlation table of canopy traits (BLUPS) for the average effect of the hybrids with Female 2.

Trait	S-pre		AUC-pre		Adjusted AUC-pre		Max-NDVI		S-post		AUC-post		Adjusted AUC-post		LSN		Yield	
	Cor.	Sig.	Cor.	Sig.	Cor.	Sig.	Cor.	Sig.	Cor.	Sig.	Cor.	Sig.	Cor.	Sig.	Cor.	Sig.	Cor.	Sig.
S-pre	1	—	0.72	∗∗∗	-0.22	∗∗	0.78	∗∗∗	-0.47	∗∗∗	0.57	∗∗∗	0.04	ns	0.19	∗	0.08	ns
AUC-pre	0.72	∗∗∗	1	—	0.16	∗	0.79	∗∗∗	-0.45	∗∗∗	0.56	∗∗∗	0.02	ns	0.13	ns	0.20	∗∗
Adjusted AUC-pre	-0.22	∗∗	0.16	∗	1	—	—	—	0.23	∗∗	-0.43	∗∗∗	-0.22	∗∗	-0.07	ns	0.15	∗
Max-NDVI	0.78	∗∗∗	0.79	∗∗∗	—	—	1	—	-0.56	∗∗∗	0.79	∗∗∗	—	—	0.14	∗	0.11	ns
S-post	-0.47	∗∗∗	-0.45	∗∗∗	0.23	∗∗	-0.56	∗∗∗	1	—	-0.03	ns	0.60	∗∗∗	-0.56	∗∗∗	0.23	∗∗∗
AUC-post	0.57	∗∗∗	0.56	∗∗∗	-0.43	∗∗∗	0.79	∗∗∗	-0.03	ns	1	—	0.71	∗∗∗	-0.26	∗∗∗	0.28	∗∗∗
Adjusted AUC-post	0.04	ns	0.02	ns	-0.22	∗∗	—	—	0.60	∗∗∗	0.71	∗∗∗	1	—	-0.57	∗∗∗	0.33	∗∗∗
LSN	0.19	∗	0.13	ns	-0.07	ns	0.14	∗	-0.56	∗∗∗	-0.26	∗∗∗	-0.57	∗∗∗	1	—	-0.28	∗∗∗
Yield	0.08	ns	0.20	∗∗	0.15	∗	0.11	ns	0.23	∗∗∗	0.28	∗∗∗	0.33	∗∗∗	-0.28	∗∗∗	1	—

Cor.: correlation; sig.: significance. Significance tested in a regression analysis. Significance level: ^∗∗∗^*P* < 0.001, ^∗∗^*P* < 0.01, ^∗^*P* < 0.05, ns: *P* > 0.05. S-pre: slope preanthesis; AUC-pre: total area under the curve preanthesis; adjusted AUC-pre: adjusted area under the curve preanthesis for maximum greenness; max-NDVI: maximum normalized difference vegetation index; S-post: slope postanthesis; AUC-post: total area under the curve postanthesis; adjusted AUC-post: adjusted area under the curve postanthesis for maximum greenness; LSN: leaf senescence.
